# Mucus mediated protection against acute colitis in adiponectin deficient mice

**DOI:** 10.1186/s12950-015-0079-y

**Published:** 2015-04-29

**Authors:** Kamaljeet Kaur, Arpit Saxena, Bianca Larsen, Samantha Truman, Nathan Biyani, Emma Fletcher, Manjeshwar Shrinath Baliga, Venkatesh Ponemone, Shweta Hegde, Anindya Chanda, Raja Fayad

**Affiliations:** Department of Exercise Science, University of South Carolina, Columbia, SC 29208 USA; Department of Research, Father Muller Medical College, Kankanady, Bangalore, Karnataka 560078 India; Fortis-Totipotent RX Centre for Cellular Medicine, Delhi, 122002 India; Department of Environmental Health Sciences, University of South Carolina, Columbia, SC 29208 USA; Center for Colon Cancer Research, University of South Carolina, Columbia, SC 29208 USA; Arnold School of Public Health, Applied Physiology Division, University of South Carolina, 921 Assembly St. room 303B, Columbia, SC 29208 USA

**Keywords:** Adiponectin, Colitis, Inflammation, Mucin

## Abstract

**Background:**

Acute ulcerative colitis is an inflammation-driven condition of the bowel. It hampers the general homeostasis of gut, resulting in decreased mucus production and epithelial cell renewal. Adiponectin (APN), an adipocytokine, is secreted by the adipose tissue and has been debated both as a pro-inflammatory or anti-inflammatory protein depending on the disease condition and microenvironment. The present study delineates the role of APN depletion in mucus modulation in a model of acute colitis.

**Methods:**

APNKO and C57BL/6 (WT) male mice were given 2% DSS *ad libidum* for 5 days in drinking water, followed by normal drinking water for the next 5 days. Hematoxyline-eosin and Alcian Blue staining was used to observe the general colonic morphology and goblet cell quantification respectively. Protein expression levels were quantified by Western blot for MATH1, Hes1, MUC2 and MUC4. ELISA was used to study the levels of TNF-α, IL-6 and IL-1β.

**Results:**

APNKO mice showed significantly higher goblet to epithelial cell ratios, lower pro-inflammatory cytokines and higher MUC2 levels as compared to the WT mice. The protein expression levels for the mucin MUC2 supported the histopathological findings. An increase in colon tissue-secreted levels of pro-inflammatory with a reduction in anti-inflammatory cytokines in presence of APN support the pro-inflammatory role of APN during acute inflammation.

**Conclusion:**

Absence of APN is protective against DSS-induced acute colonic inflammation by means of reducing colon tissue-secreted pro-inflammatory cytokines, modulating goblet and epithelial cell expressions, and increasing the levels of secretory mucin MUC2.

## Background

Inflammatory Bowel Disease (IBD) is a lifestyle disease that encompasses many inflammatory disorders, particularly Ulcerative Colitis (UC) and Crohn’s disease. Factors such as the genetic make-up, gut flora, environment and lifestyle play an important role in deciding the susceptibility to IBD [1]. Acute UC is caused by underlying events of altered immune response and consequent epithelial cell damage [2].

Adiponectin (APN), an adipocytokine produced from the adipose tissue, is now known for its anti-inflammatory, immunomodulatory and insulin-sensitizing effects [3,4]. It is a cytokine, which is present at the concentration of 5–20 μg/ml in the human blood [5]. In its monomeric form, APN contains a collagenous and a globular domain, and forms a basic homotrimeric structure via non-covalent interactions of its collagenous domains [6]. This trimeric structure can further polymerize to form APN molecules of various molecular weights [7]. It has been shown that APN may play a role in suppressing colitis [8]. It has been found to be anti-inflammatory in immune and endothelial cells [9,10]. Clinically, hypertrophied mesenteric adipose tissue of patients with Crohn’s disease is capable of secreting high levels of APN and its expression level inversely correlated with disease severity, suggesting a potential role of APN in the suppression of colitis [11]. APN bears a protective role against DSS-induced murine colitis and an anti-inflammatory effect on intestinal epithelial cells [12]. Administration of DSS delayed recovery from severe colitis and a reduced survival rate in APN knock out (KO) mice as compared to the C57BL/6 wild type (WT) mice. Furthermore, APN acts as an anti-inflammatory molecule for immune cells and endothelial cells. However, APN can act as an anti-inflammatory as well as a pro-inflammatory molecule in different settings [13]; for example, apart from the aforesaid anti-inflammatory effects of APN, it also exhibits a pro-inflammatory role in the synovial fibroblasts via production of pro-inflammatory cytokine IL-6 and matrix metalloproteinase-1, and in colonic epithelial cell line (HT-29) where it stimulates proliferation and secretion of cytokines such as IL-8 [14,15]. All the above mentioned statements show that whether the role of APN is anti- or pro-inflammatory, is determined to a great extent on cell-type and microenvironment. This observation can be explained in two ways; firstly, APN has an ability to bind lipopolysaccharides which confers a resistance on it for bacterial antigens [16], and/or secondly, APN may have a possible interaction with mucin proteins, which might alter the protective function of mucus in the colon [17]. Interestingly, it has been shown that APN deficiency confers a protective role against DSS-induced inflammation [8].

Potential contributors to intestinal homeostasis include intestinal flora, epithelial cell layer, host’s immune system components and the mucus layer. The colon lining is composed of simple columnar epithelium shaped into straight tubular crypts. The stem cells residing at the base of the crypt differentiate into epithelial and mucus-producing goblet cells. The mucus layer that overlies the epithelial cell layer is a part of innate immunity and serves the purpose of protection, lubrication and transport inside the colon. Mucin, a major component of mucus, is composed of highly glycosylated proteins forming a gel-like protective covering over the epithelial cell layer [18]. Secretory mucins are produced from the apical part of the goblet cells [19]. Although the goblet cells are present throughout the intestine, but majority resides in the colon, owing to a greater need for lubrication and transport, besides the general purpose of protection. The mucus layer protects the underlying epithelial layer from components of the host’s immune system. The erosion of this layer is one of the hallmarks of IBD [13]. Once the underlying epithelial cell layer gets exposed to the outer environment comprising the gut flora, luminal antigens and inflammatory cells, a vicious cycle of erosion, inflammation and proliferation of cells starts.

The intestinal homeostasis is also regulated by the mucins produced by the goblet cells of the gut lining. The regulation of mucin genes is involved with the dynamic nature of the mucus layer [19,20]. There occur two kinds of mucins in the gut: secretory and membrane-bound. Less is known about their differential roles during acute inflammation. Although some studies have yielded evidence that secretory mucins such as MUC2 may be induced as a result of inflammatory stimulation [21] and that mice deficient in the MUC2 spontaneously develop enterocolitis [22], the same may not apply to the membrane-bound mucins like MUC4.

Changes in the goblet cell numbers and mucus layer of the colon have been associated with intestinal inflammation, along with mucin misfolding [23]. It has also been shown that the Notch signaling pathway controls the expression of downstream signaling genes, namely Hes1 (Hairy and Enhancer of split type-1 protein) and Math1 (a transcriptional factor for the development of secretory phenotype of cells) [24]. The Hes1 gene bears an antagonistic effect but is essential for Math1 gene expression via the Notch signaling pathway. Hes1 directly interacts with the 5′ promoter region of Math1 gene to inhibit goblet cell differentiation [25].

The purpose of our study is to establish the pro-inflammatory role of APN in acute inflammation through mucus modulation. We hypothesized that the absence of APN is protective during acute inflammation. We used DSS model to induce acute inflammation in the experimental mice.

## Materials and methods

### Animals and experimental groups

Six to eight weeks old APNKO and C57BL/6 male mice were housed in a conventional animal room and treated for experimentation in the Animal Resource Facility at the University of South Carolina, Columbia. All animal procedures were approved by the Institutional Animal Care and Use Committee (IACUC) prior to the start of the study. The mice were subjected to a 12:12 hour light–dark cycle in low-stress conditions (22°C, 50% humidity and low noise) with access to food (Purina chow) and water *ad libitum*. The care and treatment of the animals followed the guidelines provided by the Institutional Animal Care and Use Committee at the University of South Carolina. APNKO mice were assigned to 1) Control, 2) DSS, 3) APN and 4) DSS + APN groups, while WT mice were assigned to 1) Control and 2) DSS groups (n = 5 mice per group). There was no significant difference between the body weights of APNKO and WT mice as measured at the beginning of the study.

### Induction of acute inflammation, APN administration and clinical score

Acute inflammation was induced in APNKO and WT mice assigned to the DSS group. These mice received 2% dextran sodium sulfate (DSS) (MW 36,000-50,000 MP Biochemicals) in drinking water for 5 days followed by normal drinking water for next 5 days, which constitutes a single cycle of DSS administration and represents the induction of acute inflammation in the gut. Treatment groups were administered either 1.5 mg/kg bodyweight of recombinant murine APN (Creative Biomart, New York) or the same amount of PBS intraperitoneally depending on the treatment group every alternate day during the 10-day period. Clinical scores were based on weight, diarrhea and fecal hemoccult on a quantifiable scale of 12 as follows: (i) a score of 1, 2, 3, or 4 was assigned for 0-5%, 6-10%, 11-15%, 16-20% and more than 20% weight loss respectively, (ii) a score of 0, 2 or 4 was assigned for well-formed fecal pellets, pasty and semi-formed fecal pellets and liquid stools that adhere to the anus, respectively, and, (iii) a score of 0, 2 or 4 was assigned to absence of blood in stools, positive hemoccult and gross bleeding respectively. Clinical score was measured every alternative day for the 10 day study period starting from day 0.

### Blood and tissue collection

All mice were euthanized by cervical dislocation on day 11. The colon obtained from the mice was flushed with PBS containing 1% solution of 5,000 IU/ml penicillin and 5,000 μg/ml streptomycin (CELLGRO). Blood and colon tissue samples were harvested from the experimental animals following euthanasia. Blood was obtained from the inferior vena cava was centrifuged at 10,000 rpm for 15 minutes. The sera were isolated and stored at −20°C until any experimentation. 2 mm^2^ excisions obtained from the distal parts of the mouse colon tissues were stored in 10% formalin for 24 hours and thereafter were put in 70% ethanol before being processed for paraffin embedding for microtome-assisted preparation of histological slides. 1 cm sections of the distal colon were added to 1 ml RPMI medium containing 1% penicillin (5,000 IU/ml) and streptomycin (5,000 μg/ml) and were incubated at 37°C (5% CO_2_) for 24 hours to obtain colon tissue secreted cytokines into the medium. The medium was centrifuged at 2,500 rpm for 15 minutes at 4°C and the supernatant was stored at −20°C until further experimentation. The rest of the colon tissues were stored at −80°C for protein expression studies.

### Histology

The general histology of the colon tissue samples was confirmed using hematoxylin and eosin staining. Alcian Blue and Neutral Fast Red staining was used for quantifying goblet/epithelial cell quantification among different experimental groups of colon tissues. Goblet (stained blue with Alcian Blue) and epithelial cells (stained pink with Neutral Fast Red) were counted using ten crypts per colon tissue section and five tissue samples from each experimental animal.

### Protein analyses

Colon tissue frozen at −80°C were homogenized in RIPA buffer supplemented with protease and phosphatase inhibitors (SIGMA). Homogenate was then centrifuged at 10,000 rpm for 15 minutes and supernatant was collected for protein analysis. Protein concentration in the supernatant was determined using Bradford protein assay. With the protein samples Western Blot was performed according to the protocol described previously [13] using primary antibodies against Hes1, Math1, MUC2 and MUC4 (Cell Signaling Technology). For quantitative comparison of the protein levels among the samples, a densitometry analysis was performed on the protein bands was performed using Image J software.

### Enzyme Linked Immunosorbent Assay (ELISA)

Spontaneous secreted cytokines was measured from the tissue incubated in the RPMI medium for 24 hours at 37°C. The media was collected and centrifuged at 2500 rpm for 16 minutes. Pellet was discarded and the supernatant was isolated. IL-6, IL-1β and IL-10 cytokines levels will be measured by using BD OptEIA ELISA kit obtained from BD biosciences and normalized by total protein content estimated by using standard Bradford assay procedure. Serum APN was also measured using standard ELISA procedure using hit from R&D systems.

### Statistical analysis

Two-way and One-way analysis of variance (ANOVA) was used to analyze the data with Tukey post hoc-analyses. A p-value < 0.05 was considered statistically significant. All the statistical analyses were done by using SigmaStat 3.5 (SPSS, Chicago, IL).

## Results

### Presence of APN is associated with higher clinical scores in experimental animals

**Figure 1:**
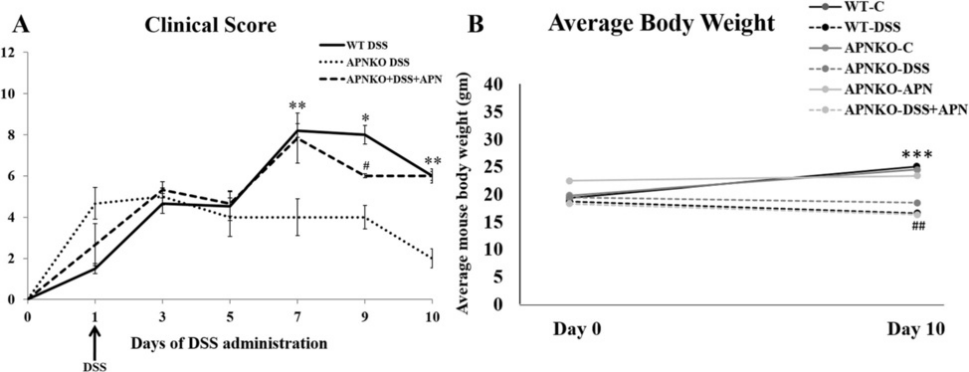
Clinical score and weight profile. **(A)** Clinical scores for DSS-treated WT, and DSS- and/or APN- treated APNKO mice during the 10 days of study. Weight loss, diarrhea and fecal hemoccult were used as parameters to calculate the clinical score; **(B)** Average weight for all experimental groups on day 0 and day 10 of the study. *p < 0.05 (vs APNKO-DSS); **p < 0.05 (vs WT-DSS, APNKO-DSS); #p < 0.04 (vs APNKO-DSS); ***p < 0.01 (Control vs DSS, WT and APNKO); ^**##**^p < 0.04 (APNKO-APN vs APNKO-DSS + APN).

We found that APN deficiency was protective against DSS-induced acute colitis in the experimental animals. DSS-administered APNKO animals had the lowest clinical score throughout the ten days while the WT-DSS group of animals exhibited the highest clinical score (p < 0.05) (Figure 1A). An intermediate clinical score was observed with the APNKO-DSS group that was administered with APN. Mice weight (gm) was determined on day 0 and 9 for all the groups. No significant difference was found among the groups on day 0 of the study. However, we found a significant reduction in the average mice weight with DSS treatment between APNKO-DSS + APN and APNKO-APN (p < 0.04), APNKO-DSS and APNKO-C (p < 0.01), and WT-DSS and WT-C (p <0.01) (Figure 1B) on day 9.

### Lower serum APN is associated with DSS administration

**Figure 2:**
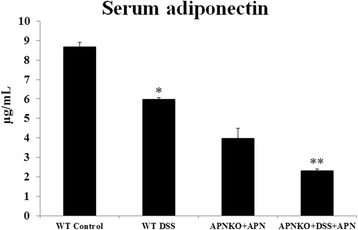
Serum APN measurements**.** ELISA data showing serum APN levels in four experimental groups. *p < 0.05 (vs WT-C); **p < 0.03 (vs APNKO-APN).

As a first step to test our hypothesis that the absence of APN is protective during acute inflammation, we initially proceeded to quantify the serum APN level using ELISA for all the experimental groups that were investigated in this study. Our data (Figure 2) shows that the serum APN levels were significantly lower in APNKO-DSS + APN and WT-DSS groups than their non-DSS controls, APNKO-APN and WT-C groups respectively (p < 0.05) (Figure 2). APNKO-C control group showed no serum APN (data not shown).

### Colon morphology was altered following APN administration during DSS-induced acute colitis

**Figure 3:**
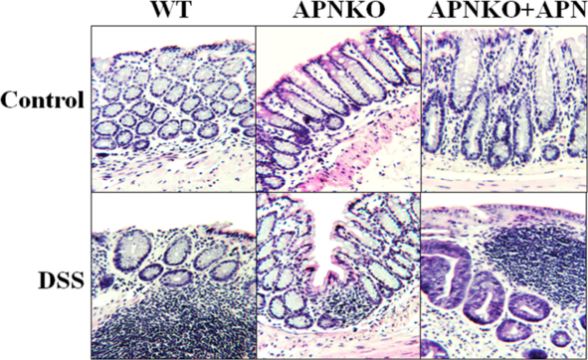
Colon histopathology. Hematoxylin and eosin stained colon tissues taken from mice belonging to different treatment groups, showing their respective histopathology following APN administration.

Colon morphology was altered following APN administration during DSS-induced acute colitis. Following the DSS treatment, we administered APN to the animals in order to specify its role in modulating inflammation in response to the DSS-induced colon insult. We found that absence of APN was protective against DSS-induced colonic insult as was observed by inflammatory cell infiltration. The APNKO-DSS + APN and WT-DSS groups revealed the maximum amount of inflammation and aberrant colonic crypts (Figure 3).

### Increased goblet to epithelial cell ratio in APNKO with acute inflammation

**Figure 4:**
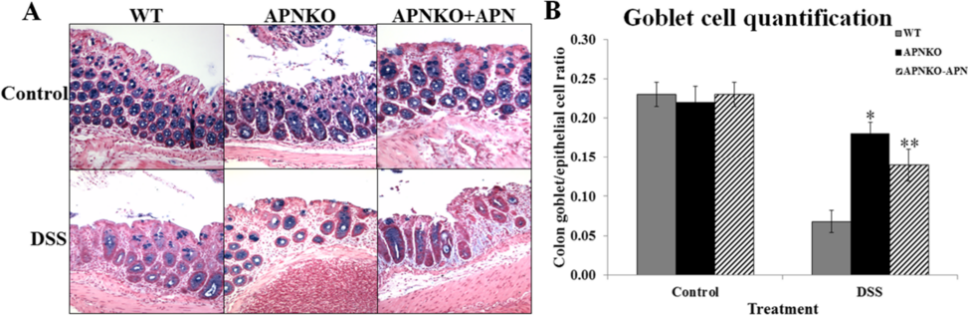
Colon goblet and epithelial cell quantification. Figure illustrating **(A)** Alcian Blue and Nuclear Fast Red stained colon tissues taken from mice belonging to different treatment groups, showing numbers goblet cells relative to epithelial cells; and **(B)** quantification of goblet and epithelial cells in colon, following APN administration. *p < 0.01 (vs WT-DSS); **p < 0.01 (vs APNKO-DSS).

The differential numbers of goblet and epithelial cells within the colon crypts were quantified histologically. Within a crypt, the goblet cells were stained blue with Alcian Blue stain while the epithelial cells were stained pink with Nuclear Fast Red (Figure 4A). Goblet to epithelial cell ratio was found to be significantly higher (p < 0.001) in the APNKO mice as compared to WT mice in DSS treatment group. Significantly lower (p < 0.01) goblet to epithelial cell ratio was found in WT mice given DSS treatment as compared to the control WT mice. No significant difference was observed in the control group (Figure 4B).

### Math1/Hes1 expression level ratios increased in the APNKO genotype with acute inflammation

**Figure 5:**
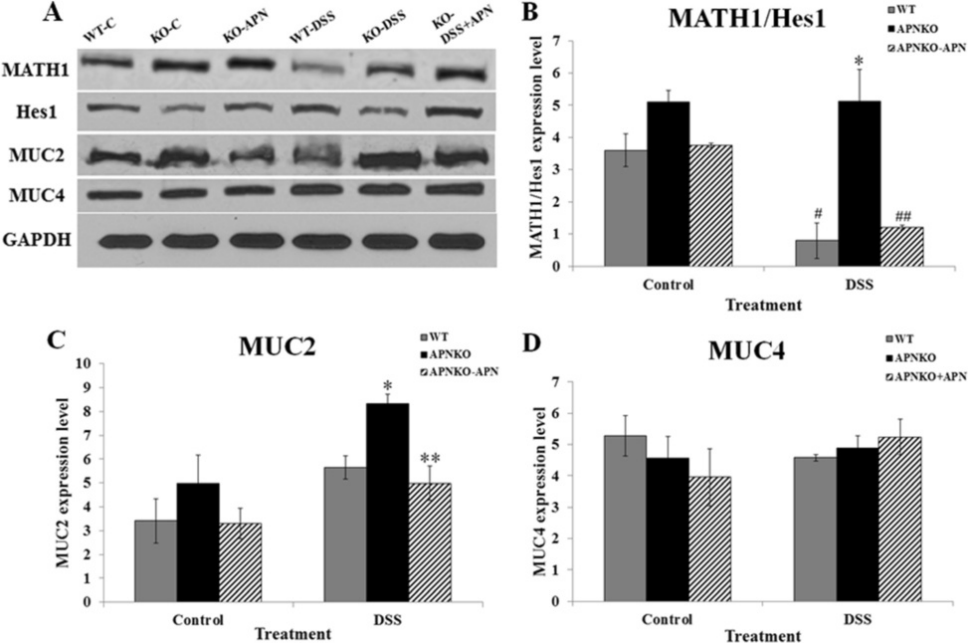
Protein expression profile. **(A)** Respresentative Western blot image of the expression levels of MATH1, Hes1, MUC2, MUC4 and GAPDH. **(B-D)** Protein expression levels of MATH1/Hes1, MUC2 and MUC4 in colon tissues of experimental mice. *p < 0.04 (vs WT-DSS); **p < 0.04 (vs APNKO-DSS or WT-DSS); #p < 0.04 (vs WT-C); ##p < 0.04 (vs APNKO-DSS or APNKO-APN).

Expression of specific proteins related to mucus production was studied by Western blot (Figure 5A). Math1 and Hes1 genes were studied for their relative expression levels as a possible mechanism behind the modulation of the differential expression of goblet and epithelial cells in the colon. A significantly higher (p < 0.01) Math-1/Hes-1 ratio was found in the DSS-treated APNKO mice as compared to their WT counterparts. Moreover, the DSS-treated APNKO mice showed higher (p < 0.02) Math-1/Hes-1 ratio than the APNKO mice in control group (Figure 5B). No significant difference was found between other treatment groups.

### APN may exert its effects on mucus production by increasing secretory mucins but not membrane-bound mucins

We aimed at quantifying the expression levels of secretory mucin MUC2 and membrane-bound mucin MUC4 in order to characterize the role APN in modulating either or both kinds of mucins during acute inflammation. We found that APN deficiency proved to be protective in DSS-induced acute inflammation as seen through increased MUC2 expressions, which were significantly higher (p < 0.04) in the APNKO-DSS groups as compared to WT-DSS and APNKO-DSS + APN groups (Figure 5C). MUC2 protein expression levels were consistent with our goblet cell staining and quantification data suggesting that MUC2 can be a major secretory protein produced during the acute phases of inflammation in the gut. However, the results obtained for MUC4 protein expression were not similar to MUC2 expression. No significant difference in MUC4 protein expression was observed among the experimental groups following DSS and/or APN administration (Figure 5D) indicating a possibility of a major role being played by the secretory proteins in protection against inflammation than by membrane-bound mucins.

### APN proved to be pro-inflammatory in acute inflammation by eliciting a pro-inflammatory cytokine production

**Figure 6:**
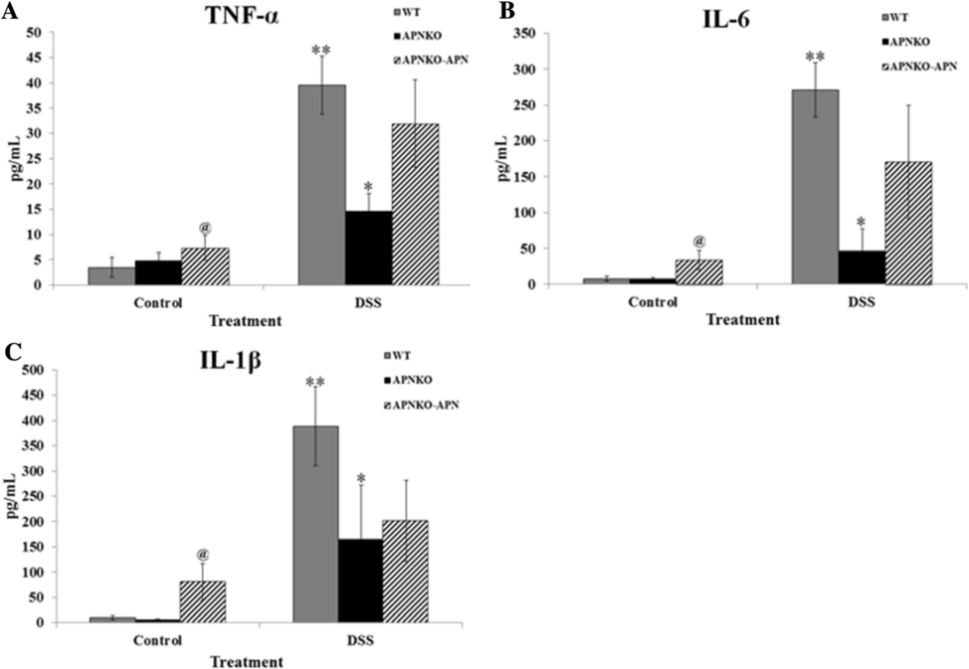
Colon tissue-secreted cytokines. The figure illustrates ELISA profiles of cytokines **(A)** TNF-α, **(B)** IL-1β and **(C)** IL-6 secreted from the colon tissue of experimental mice belonging to different treatment groups. *p < 0.05 (vs APNKO-C or WT-DSS); **p < 0.03 (vs WT-C); @p < 0.05 (vs APNKO-C).

Numerous studies pertaining to the role of APN in different disease conditions have reported it as both an anti- and a pro-inflammatory molecule. We measured the levels four different cytokines from colon culture supernatents to establish the role of APN in dictating inflammatory response through the modulation of cytokines. Levels of colon-secreted IL-1β, IL-6 and TNF-α were increased following DSS administration as compared to the control (Figure 6A-C). Interestingly, the levels remained the lowest for the APNKO-DSS group. The levels of the pro-inflammatory cytokines significantly increased (p < 0.05) once APN was re-administered to the APNKO-DSS group, establishing the pro-inflammatory response elicited by APN on the colon.

## Discussion

APN has previously been demonstrated as a pro-inflammatory molecule [8], where effects of its absence using APNKO mice were explored in acute inflammation. The present study focused on elucidating the role of exogenously administered APN during acute inflammation in mice during DSS-induced acute phase of inflammation. It was found that lack of APN is beneficial during acute inflammation, as indicated by the lowest clinical score exhibited in APNKO group as compared to APNKO-APN and WT groups during acute inflammation (Figure 1A). The morphological responses of the colon tissue to induced acute inflammation (Figure 3) support the clinical scores obtained, which also confirm previous findings in similar settings [8], where APN was studied for its binding abilities to growth factors and induction of its receptors during acute inflammation.

An interesting observation in this study was a significant reduction in serum APN levels upon DSS administration in both WT and APNKO + APN mice as revealed in our ELISA data in Figure 2. We speculate that this reduction of serum APN could result from the decrease in weights upon DSS induced inflammation (as shown in Figure 1B); the severity of inflammation can result in this weight loss from lower food consumption and greater energy expenditure in repair mechanism. It is possible that a decrease in adipose tissue during weight loss upon DSS administration results in reduction in serum APN; since adipose tissue are the primary site for APN production, a decrease in adipose tissue would result in a drop in serum APN as well.

Our ELISA data also indicated that addition of APN to APNKO mice (in both APNKO-APN and APNKO-DSS + APN groups) could not bring up the levels of serum APN levels to those in the WT-C group. The lack of any significant differences in goblet cell numbers between the WT, APNKO and APNKO-APN control groups indicates that APN has no adverse effect in the absence of DSS. However as expected from our ELISA data, that external administration of APN to APNKO could not completely reverse goblet cell/epithelial cell ratio (Figure 4). Although the observations do not undermine the protective role of absence of APN during acute inflammation, they do suggest that external administration of APN is not as effectively absorbed into blood stream as that secreted from the adipose tissue under normal healthy conditions.

Our study also demonstrated mucus modulation as another aspect of the protection rendered by absence of APN against acute inflammation. A higher number of goblet cells quantified histologically as compared to the number of epithelial cells found in the colon crypts in the APNKO genotype indicated a mucus-mediated protective mechanism during acute inflammation (Figure 4A, B).

Epithelial to goblet cell differentiation has been well associated with the expression levels of the Hes1 and Math1 genes. The two genes are part of and regulated by the Notch signaling pathway. The upregulation of the Notch signaling pathway brings about a downregulation of Math1 mRNA levels whereas an upregulation of Hes1 gene expression. The two genes interact directly with each other, such that an increase in the *Math1* expression levels results in an increased goblet cell expression. *Math1* mutants are known to lack all kinds of secretory cells but retain the absorptive cells [26] and the cell fate is determined with the modulation of the Notch signaling cascade. Interestingly, a high level of Math1 protein expression correlates positively with increased goblet to epithelial cell ratio, which is supported by our data (Figure 4A, B), which further supports our hypothesis that an increased protection from DSS induced acute inflammation in the absence of APN might be dictated by the upregulation of Math1 expression, hence leading to increased goblet cells and mucus secretion. The upregulation of Hes1 on the other hand, has been linked to the differentiation of progenitor stem cells into epithelial cells in the gut. Although Hes1 binds to the promoter region of Math1 and brings about its inhibition, we did not see a reciprocal effect in the expression levels of Math1 and Hes1 genes in the present study. These observations may represent a protective adaptation in the gut from DSS insult, where there is an increase in the epithelial cell proliferation, which could be directed by increased Hes1 gene expression, with a simultaneous upregulation of Math1 expression, bringing about an increase in the goblet to epithelial cell ratio. Our data pertaining to Math1 to Hes1 ratios among the experimental groups depicts a significantly higher Math1 to Hes1 expression ratio in the DSS-treated APNKO mice as compared to their WT counterparts. The ratio was also significantly higher in the DSS-treated APNKO mice as compared to the APNKO mice without treatment (Figure 5B). A simultaneous increase in Math1 and Hes1 protein levels may serve as a protective mechanism in response to the earliest phases of inflammation.

An increase in the levels of MUC2 in the absence of APN indicates that APN might exert its effects through its interactions through secreted mucins (Figure 5C). Previously, studies have demonstrated that MUC2 serves as an important mucin in the gut and indicates the cellular status of the colon lining [17,27]. However, the levels of MUC4, an important membrane-bound mucin, remained non-significant between the different experimental groups of our study (Figure 5D), indicating that it is the secreted mucins that might play a role in the protective effects of APN during acute inflammation. The exact nature of this interaction and its potential downstream signaling is still unclear.

The fact that APN production is reduced in conditions such as Type-2 diabetes, metabolic syndrome and cardiovascular disease [28], and that APN prevents atherosclerosis, fatty liver disease and hepatic fibrosis [29-31], makes it an anti-inflammatory molecule. Its anti-inflammatory properties have also been demonstrated *in vitro* [10]. However, studies have also characterized APN as possessing pro-inflammatory roles, mostly *in vitro* [15] but also *in vivo* [8]. The pro-inflammatory role of APN has been attributed to its binding to lipopolysaccharide [16] and an apparent modulation of tolerance to bacterial antigens and/or its binding to growth factors that induce pro-inflammatory changes in the gut [8]. A major pro-inflammatory effect of APN in our study can be seen as quantified by the upregulation of major pro-inflammatory cytokines – IL-1β, IL-6 and TNF-α (Figure 6A-C). These results are consistent with previous *in vitro* findings that relate APN with the NF-ĸB activation [28,32]. IL-6 level was found to be augmented in the presence of APN as measured in colon culture supernatants, which consolidates our previous findings [8]. An increase in TNF-α secretion from colon tissues in the presence of APN reflects the pro-inflammatory nature of APN in case of acute inflammation. Since TNF-α has been implicated in many inflammatory conditions [33], it is likely that APN exerts its pro-inflammatory effects through the activation of TNF-α. Also, it has been shown that IL-1β is upregulated especially during acute inflammation [34]. Our findings on colon secreted IL-1β revealed a reduction of its levels in the absence of APN, further indicating that the APN is pro-inflammatory during acute inflammation.

## Conclusion

The present study demonstrated the pro-inflammatory role of APN during acute colitis. The presence of APN acts as an inducer of pro-inflammatory cytokines during acute phase of inflammation. Mechanistically, presence of APN not only mediates an inflammatory response through a localized secretion of cytokines from the colon, but also leads to a reduction in the mucus levels through its modulation of mucin-regulatory genes and especially through reductions in the secretory mucin MUC2. Further studies are needed to elucidate mechanistically the direct effect of APN on mucus production during acute inflammation.

## Abbreviations

APN: Adiponectin
APNKO: Adiponectin-knockout
WT: Wild-type
C: Control
DSS: Dextran sodium sulphate
IACUC: Institutional Animal Care and Use Committee
ELISA: Enzyme-linked immunosorbent assay
ANOVA: Analysis of variance
